# Supplementary material for: All-at-once RNA folding with 3D motif prediction framed by evolutionary information
Source: Nat Methods. 2025 Oct 3;22(10):2094–106. doi: 10.1038/s41592-025-02833-w (PMC12510878; doi:10.1038/s41592-025-02833-w)
Supplement: Supplementary file 3 — Source code to run CaCoFold-R3D. Data and results. [file 41592_2025_2833_MOESM3_ESM.gz › supplemental_material/results/CaCoFold-R3D/CaCoFold-R3D_Rfam_vshuffle/RF01986_ZEB2_AS1_3.R2R.sto.pdf]

RF01986_ZEB2_AS1_3

5′ ‒ Y C C ● C ● C Y Y C Y C C ● ● ● C C C C C C C ○ ○ ○ ○ ○ ○ ○ C C G U C A A G C C U U U G G C A U C A U U A U C C U C A U C A C U A A A U C C U A C A G A G U A C ● G G G C G G A A A A C G G U G C A C A C
